# Systemic chromosome instability in Shugoshin-1 mice resulted in compromised glutathione pathway, activation of Wnt signaling and defects in immune system in the lung

**DOI:** 10.1038/oncsis.2016.56

**Published:** 2016-08-15

**Authors:** H Y Yamada, G Kumar, Y Zhang, E Rubin, S Lightfoot, W Dai, C V Rao

**Affiliations:** 1 Center for Cancer Prevention and Drug Development, Department of Medicine, Hematology/Oncology Section, University of Oklahoma Health Sciences Center (OUHSC), Oklahoma City, OK, USA; 2 Department of Pathology, OU Medical Center, Oklahoma City, OK, USA; 3 Department of Environmental Medicine, New York University Langone Medical Center, Tuxedo, NY, USA

## Abstract

Mitotic error-mediated chromosome instability (CIN) can lead to aneuploidy, chromothripsis, DNA damage and/or whole chromosome gain/loss. CIN may prompt rapid accumulation of mutations and genomic alterations. Thus, CIN can promote carcinogenesis. This CIN process results from a mutation in certain genes or environmental challenge such as smoking, and is highly prevalent in various cancers, including lung cancer. A better understanding of the effects of CIN on carcinogenesis will lead to novel methods for cancer prevention and treatment. Previously Shugoshin-1 (Sgo1^−/+^) mice, a transgenic mouse model of CIN, showed mild proneness to spontaneous lung and liver cancers. In this study, adoptive (T/B-cell based) immunity-deficient RAG1^−/−^ Sgo1^−/+^ double mutant mice developed lung adenocarcinomas more aggressively than did Sgo1^−/+^ or RAG1^−/−^ mice, suggesting immune system involvement in CIN-mediated lung carcinogenesis. To identify molecular causes of the lung adenocarcinoma, we used systems biology approach, comparative RNAseq, to RAG1^−/−^ and RAG1^−/−^ Sgo1^−/+^. The comparative RNAseq data and follow-up analyses in the lungs of naive Sgo1^−/+^ mice demonstrate that, (i) glutathione is depleted, making the tissue vulnerable to oxidative stress, (ii) spontaneous DNA damage is increased, (iii) oncogenic Wnt signaling is activated, (iv) both major branches of the immune system are weakened through misregulations in signal mediators such as CD80 and calreticulin and (v) the actin cytoskeleton is misregulated. Overall, the results show multi-faceted roles of CIN in lung carcinoma development in Sgo1^−/+^ mice. Our model presents various effects of CIN and will help to identify potential targets to prevent CIN-driven carcinogenesis in the lung.

## Introduction

Aneuploidy has been predicted to cause cancer.^1, 2^ Aneuploid cells are created through genomic instability, which has two major modes, mitotic error-mediated chromosome instability (CIN) and DNA metabolism-mediated microsatellite instability, which are not mutually exclusive. CIN can lead to a variety of outcomes in post-mitotic cells, including tetraploidy, chromothripsis, DNA damage and aneuploidy.^2, 3^ Further, the gain of oncogenes or loss of tumor suppressors may result. Thus, CIN can serve as a mutator.^4^ In addition, CIN can produce DNA damage and facilitate mutagenesis.^5^ Micronuclei formation and chromothripsis may also occur, leading to extensive genome shuffling and mutation.^6, 7^ Thus, CIN is mutagenic on a cellular level. Carcinogenic environmental factors, such as hepatitis virus infection^8, 9^ and smoking,^10, 11^ can produce CIN. These cell biological observations and other evidence strongly suggest a role of CIN in carcinogenesis.

Tumor-mass sequencing data indicate that the genes that are frequently mutated in colon cancer function to prevent CIN, suggesting that progressive CIN is an integrated process in colonic carcinogenesis.^3^ Most primary lung cancers carry loss of heterozygosity, which is associated with CIN, further implicating CIN in lung carcinogenesis.^12^ CIN can be caused by mutations in various genes, many of which are *bona fide* mitotic regulators, such as mitotic spindle checkpoint components BubR1 and Mad2, the mitotic motor Cenpe, and chromosome cohesion regulators such as Sgo1. CIN model mice, transgenic mice with mutations in mitotic regulators, were created to investigate the effects of CIN on carcinogenesis *in vivo*.^13, 14, 15, 16^

The consequences of CIN and aneuploidy *in vivo* have been identified and characterized using animal models. Aneuploidy causes gross transcriptome changes^17^ and imbalances in protein generation, producing proteotoxic effects that lead to endoplasmic reticulum (ER) stress, lysosomal stress and overwhelmed autophagy in the cells.^18^ Mouse studies showed that high rates of CIN and aneuploidy do not always translate directly to high rates of cancer, and that CIN can be both oncogenic and tumor suppressing in different organs.^4, 19, 20^ Therefore, the relationship between CIN and carcinogenesis is not straightforward and is organ specific. Cell death is a determining factor. A high rate of CIN and aneuploidy can lead to cell death rather than tumorigenesis.^21^ Another possible determining factor is the involvement of immune system and its surveillance to remove aneuploid cells. G Kroemer‘s group showed that tetraploid cells express specific cell surface antigens, including calreticulin (an ‘eat me' signal), and proposed that immune surveillance specifically targets the tetraploid cells to suppress cancer.^22, 23^ Although whether immune surveillance is involved in removing CIN cells *in vivo* remains to be investigated further, there are reports supportive to the notion linking smoking, CIN and immune function, such as, tobacco smoking is known to decrease immune functions in lung,^24, 25^ and lung cancers with smoking signatures respond better to immune checkpoint PD1 inhibitor therapy.^26^

Shugoshin-1 (Sgo1) regulates chromosome cohesion and centrosome integrity.^27, 28, 29^ Sgo1 expression is abnormal in various human cancers, including colon, lung and liver cancer.^30, 31, 32, 33^ The inner centromere-Shugoshin mitotic network was shown to be dysfunctional in numerous cancers, suggesting that proper Sgo1 function is crucial to preventing CIN and cancer.^34^ To further investigate carcinogenesis for cancer prevention, we generated Sgo1^−/+^ CIN model mice, and have demonstrated the involvement of CIN in carcinogenesis in several organs, including the colon, liver and lungs.^20, 35, 36, 37^ The colon in particular showed a modified carcinogenesis profile after treatment with the carcinogen Azoxymethane.^20, 35, 37^ In the Sgo1^−/+^ CIN model mice, the lungs and liver were also prone to spontaneous carcinogenesis.^36^ Spontaneous hepatic and lung carcinogenesis were reported in other CIN models, including BubR1^−/+^ and Mad2^−/+^ mice,^16^ suggesting that these cancers may be common consequences of CIN.

CIN has shown to be at least correlational, if not causal, to carcinogenesis in the lung. Smoking increases copy number variations and CIN in lungs in humans and animal models.^38, 39^ CIN is a marker of poor prognosis for non-small cell lung cancer.^40^ Inducible Mad2 overexpression CIN model mice, which are defective in the mitotic spindle checkpoint and show CIN, demonstrated a higher rate of recurrence of lung tumor in an experimental setting in which lung tumorigenesis was at first induced through k-ras oncogene addiction then the tumors regressed by the oncogene shutdown.^41^

As above, the link between CIN and immune function has only been suggestive and the role in carcinogenesis requires further study. In this study, we generated RAG1^−/−^ Sgo1^−/+^ mice and assessed carcinogenesis to investigate the involvement of adoptive immunity and immune surveillance without or with high rate of CIN (that is, Sgo1^−/+^ background) *in vivo*. Recombination activating gene 1 (RAG1) is involved in activation of immunoglobulin V-D-J recombination. The loss (−/−) of RAG1 leads to compromised adoptive immunity,^42^ yet due to exclusive dependence on another immune system branch mediated by natural killer (NK) cells,^43^ no lung tumor proneness has been reported. We observed enhanced carcinogenesis in lungs from RAG1^−/−^ Sgo1^−/+^ mice, suggesting a link between CIN-aggravated lung carcinogenesis and adoptive immunity. To investigate the molecular link, we performed next-generation sequencing/RNAseq in lungs from RAG1^−/−^ and RAG1^−/−^ Sgo1^−/+^ mice, and identified culprit pathways responsible for the lung carcinogenesis. We validated the pathways in naive Sgo1^−/+^ mouse lungs, uncovering the multi-faceted carcinogenic effects of CIN in the lung for the first time.

## Results

### RAG1^−/−^ Sgo1^−/+^ mice showed high incidence of lung adenocarcinoma

Sgo1^−/+^ mice develop with normal appearance and with modest proneness to spontaneous carcinogenesis in the lungs and liver at 12 months of age and later.^36^ RAG1^−/−^ mice also develop normally with no apparent tumors up to 12 months, despite their compromised adoptive immunity.^42, 43^ The relatively normal phenotype of RAG1^−/−^ mice in the laboratory environment is at least partly explained by surveillance of NK cells, another major branch of the immune system.^42, 43^

**Figure 1:**
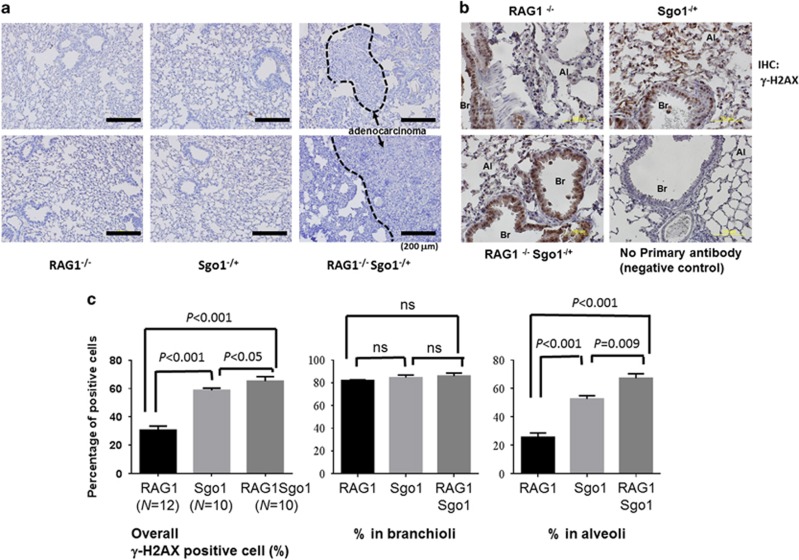
RAG1^−/−^ Sgo1^−/+^ mice developed lung adenocarcinomas. (**a**) RAG1^−/−^ Sgo1^−/+^ mice developed lung adenocarcinomas with the appearance of bronchioloalveolar in-type (that is, cuboidal cells lining the alveolar septa and projecting into alveolar spaces). RAG1^−/−^ and most Sgo1^−/+^ did not show significant histopathological changes, with some exceptions in Sgo1^−/+^ that developed adenocarcinomas in smaller sizes. (**b**) To assess DNA double-strand break, IHC for γ-H2AX was performed. Percentages for γ-H2AX positive cells were high in Branchioli, but varied between Sgo1^−/+^ and Sgo1^+/+^ backgrounds in alveoli. Al, alveole; Br, branchiole. (**c**) Rag1^−/−^Sgo1^−/+^ showed highest percentage of γ-H2AX positive cells. However, signals at branchioli showed little difference (NS, nonsignificant). The difference between Sgo1^−/+^ and Sgo1^+/+^ backgrounds were evident only in alveoli.

To assess the effect of the loss of adoptive immunity in the Sgo1^−/+^ CIN model, we generated RAG1^−/−^ Sgo1^−/+^ mice. All animals (RAG1^−/−^ (*n*=20), Sgo1^−/+^ [*n*=32], RAG1^−/−^ Sgo1^−/+^ (*n*=16)) developed and lived normally until the ages of 6 and 9 months. However, significantly more RAG1^−/−^ Sgo1^−/+^ mice (*n*=6; 37.5%) died by the 12 months end point (Table 1). The main cause of death was gross lung tumors. Six among the 10 surviving RAG1^−/−^ Sgo1^−/+^ mice (37.5%) had gross lung carcinomas at the 12 months end point (Figure 1a). Previous studies indicated a modest increase in spontaneous liver cancers^36^ and lung cancers^36^ (Table 1) in Sgo1^−/+^ mice. However, the cancers did not cause a significant increase in lethality because of the smaller size by 12 months of age. The RAG1^−/−^ background facilitated lung carcinoma development in Sgo1^−/+^ such that it manifested as lethality by 12 months of age. Gross liver tumors were not observed, although histological abnormalities such as nuclear pleomorphism (variation of nuclear size), megamitochondria and binucleation were observed in mice with Sgo1^−/+^ background. No significant inflammatory infiltrates or steatosis were appreciated in any of the sections examined. (Supplementary Figure 1). When we performed lung immunohistochemistry (IHC) for DNA double-strand break marker γ-H2AX in RAG1^−/−^Sgo1^−/+^ double mutant and compared with RAG1^−/−^ and Sgo1^−/+^ single mutants, there were clear difference in γ-H2AX staining pattern between Sgo1^−/+^ and Sgo1^+/+^. Sgo1^−/+^ background specifically showed higher γ-H2AX in alveoli, but not in branchioli (Figures 1b and c), which suggests that the branchioloalveolar-in type lung tumors may have originated from alveolar region with higher DNA damage.

### Transcriptomic differences in normal-looking lung tissues from RAG1^−/−^ and RAG1^−/−^ Sgo1^−/+^ mice

**Figure 2:**
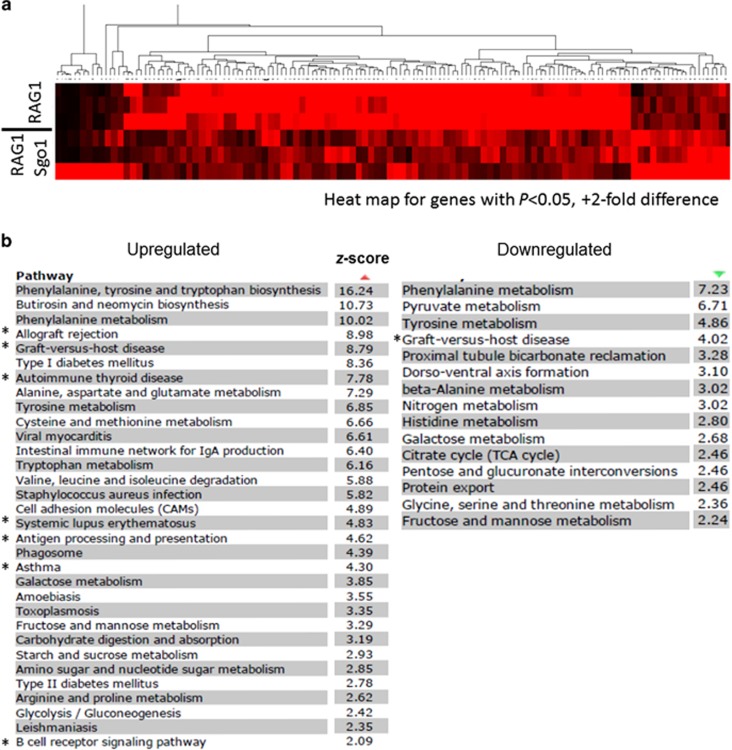
Differentially expressed pathways in normal-looking lung tissues from RAG1^−/−^ and RAG1^−/−^ Sgo1^−/+^ mice. Next-generation sequencing/RNAseq identified 72 upregulated and 81 downregulated genes in lungs of RAG1^−/−^ Sgo1^−/+^ mice compared with RAG1^−/−^ (*P*<0.05, 2-fold). (**a**) Heat map for genes with P<0.05, +2-fold difference, indicating consistency within a group and difference between two groups. The colors represent the range of gene expression. Black, a reduced expression value; red, an increased expression value. The deeper color is a higher expression values whether reduced or increased. (**b**) Pathway analysis identified most affected pathways (*z* score >2). Asterisks (*) indicate immune function-related pathways shared with previous colonic transcriptome analysis between wild-type and Sgo1^−/+^ mice.^37^

To investigate the molecular causes that led to high incidence of lung carcinomas in RAG1^−/−^ Sgo1^−/+^ mice, we performed RNAseq-transcriptome analyses using normal-looking lung tissues from RAG1^−/−^ (*N*=3) and RAG1^−/−^ Sgo1^−/+^ (*N*=3) mice. Using the data set, we identified differentially expressed genes. There were total of 153 hits with a twofold expression difference threshold, *P*<0.05; 72 upregulated genes and 81 downregulated genes (complete list in Supplementary Data). Heat map for the identified genes showed consistent differences between RAG1^−/−^ and RAG1^−/−^Sgo1^−/+^ groups (Figure 2a). Significantly affected pathways (*z* score >2) were identified, and indicated as Kyoto Encyclopedia of Genes and Genomes pathways (Figure 2b). Upregulated pathways included various amino-acid metabolism (for example, phenylalanine, tyrosine and tryptophan biosynthesis (*z* score 16.24); phenylalanine metabolism (10.02); alanine, aspartate and glutamate metabolism (7.29); tyrosine metabolism (6.85); and cysteine and methionine metabolism (6.66)), and immune function-related (for example, allograft rejection (8.98); graft-vs-host disease (8.79); autoimmune thyroid disease (7.78); systemic lupus erythematosus (4.83); and antigen processing and presentation (4.62)). Downregulated pathways included phenylalanine metabolism (7.23), tyrosine metabolism (4.86) and graft-vs-host disease (4.02). Overall, the pathway analysis indicated significant misregulations in amino-acid metabolism and immune system. Notably, some pathways were commonly found in colonic RNAseq-transcriptome analysis in our recent study:^37^ diabetes mellitus and graft-vs-host disease. The results indicated that the immune response (graft-vs-host disease) may be a pathway compromised in multiple organs in Sgo1-CIN mice.

### Sgo1^−/+^ lungs have less glutathione and more DNA damage

**Figure 3:**
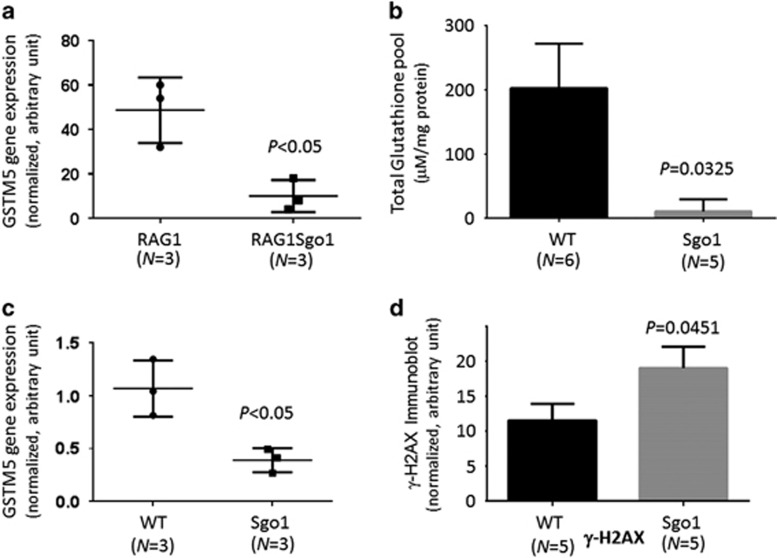
Sgo1^−/+^ lungs carried less glutathione and more DNA damage. (**a**) RNAseq analysis in RAG1^−/−^ and RAG1^−/−^Sgo1^−/+^ indicated a significant reduction in glutathione-*S*-transferase mu5 (GSTM5) expression in Sgo1^−/+^ background. (**b**) Sgo1^−/+^ lungs carry less glutathione. Wild-type and naive Sgo1 mice were compared with investigate whether the RAG1^−/−^ background affected the results. We measured the total levels glutathione (GSSG and GSH combined) in lungs of wild-type (*n*=5) and Sgo1^−/+^ (*n*=5) mice with an OxiSelect kit (Cell Biolabs Inc.). We observed a significant reduction (~18.8-fold difference) in total glutathione in Sgo1 mice, *P*<0.05. (**c**) Significant decrease in GSTM5 expression was confirmed in naive Sgo1 lung with quantitative reverse transcriptase (qRT)–PCR. (**d**) DNA damage marker γ-H2AX was quantified with quantitative immunoblot, revealing increased γ-H2AX in Sgo1^−/+^ mice lung compared with wild-type.

A noteworthy gene among the differentially expressed genes was glutathione-*S*-transferase mu5 (GSTM5) (3.37-fold down; Figure 3a). Glutathione is a major cellular anti-oxidant, and this GST downregulation would decrease the efficacy of detoxification and the oxidative stress response through glutathione conjugation. We suspected that lungs of Sgo1^−/+^ mice are vulnerable to oxidative stress because of a decrease in the glutathione-mediated protection. To test the hypothesis, we made tissue extracts from the lungs of untreated 12-month-old wild-type (*n*=6) and Sgo1^−/+^ mice (*n*=5), and measured levels of total cellular glutathione (oxidized GSSG and reduced GSH forms combined) in the lungs (Figure 3b). The wild-type lung tissues were estimated to have 18.8-fold higher total glutathione (*P*=0.0325). Quantitative real-time PCR with wild-type and Sgo1^−/+^ mice lungs confirmed that GSTM5 mRNA expression was decreased (Figure 3c). Thus, the lungs of Sgo1^−/+^ mice lack glutathione protection in two aspects; decreases in glutathione-*S*-transferase and total glutathione pool. The double decreases in GSTM5 expression and total glutathione pool would have roles in weakening resistance to oxidative stress in Sgo1^−/+^ lungs, aiding in the creation of a carcinogenesis-prone condition. To assess the degree of DNA damage, we measured γ-H2AX, which was significantly increased in Sgo1^−/+^ lungs (Figure 3d).

### Wnt signaling is activated in Sgo1^−/+^ lungs

**Figure 4:**
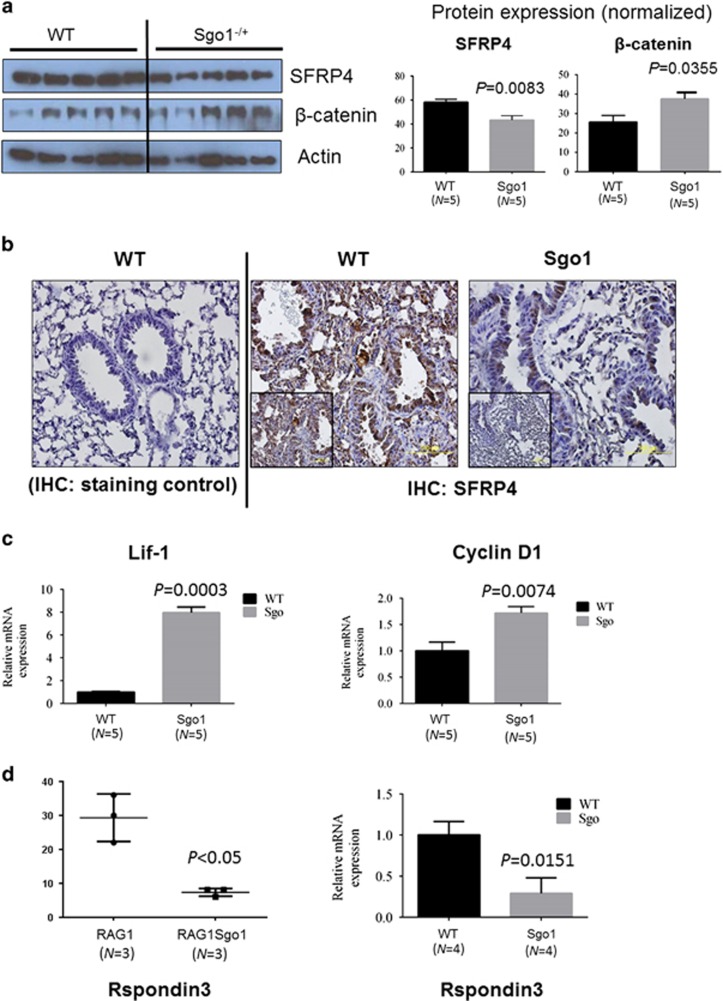
Wnt signaling was activated with various misregulated components in Sgo1^−/+^. (**a**) Wnt inhibitor SFRP4 protein was reduced, and Wnt effector β-catenin was increased. (**b**) IHC results showed that SFRP4 expression was reduced and its localization was limited in branchiole cells in Sgo1^−/+^ mice. (**c**) Wnt target genes, Lif1 and cyclin D1, were significantly upregulated, indicating Wnt signaling activation. (**d**) Wnt component Rspondin3 was downregulated both in RAG1^−/−^ and RAG1^+/+^ backgrounds, indicating Wnt misregulation in Sgo1^−/+^.

Secreted frizzled-related protein 4 (SFRP4) was among the most downregulated genes (6.05-fold downregulation). SFRP4 is a soluble modulator/inhibitor of Wnt signaling, and its underexpression activates Wnt signaling.^44^ We hypothesized that Wnt signaling is activated in Sgo1^−/+^ lungs, and tested the hypothesis by monitoring markers for Wnt signaling. As predicted from RNAseq analysis, SFRP4 protein levels were 30% lower in untreated Sgo1^−/+^ lungs (*P*<0.05, Figure 4a), and Wnt signaling effector β-catenin was 50% higher in Sgo1^−/+^ lungs (*P*<0.05). Although SFRP4 localized in both alveolar cells and branchiolar cells in control wild-type mice, in Sgo1^−/+^ mice the localization occurred mainly in branchioli cells (Figure 4b). To confirm Wnt signaling activation, we used quantitative PCR to test the expression of Wnt target genes. Wnt target genes were upregulated 8-fold in Lif1 and 1.8-fold in cyclin D1 (Figure 4c).

R-spondin3 (Rspo3) is a secreted protein that has been implicated in Wnt signaling. Rspo3 is aberrantly expressed at high levels in approximately half of all Keap1-mutated human lung adenocarcinomas, and may promote cancer aggressiveness.^45^ In zebrafish, Rspo3 knockdown activates Wnt/β-catenin signaling, which is involved in anteroposterior patterning.^46^ A 2.61-fold downregulation in Rspo3 expression was observed in the RNAseq analysis on the RAG1^−/−^ background, which was confirmed in quantitative RT–PCR in wild-type and Sgo1^−/+^ (Figure 4d). These findings further demonstrate misregulation in Wnt signaling in normal-looking lungs on the Sgo1^−/+^ background.

### Immunomodulator proteins CD80 and calreticulin are downregulated in Sgo1^−/+^ lungs

**Figure 5:**
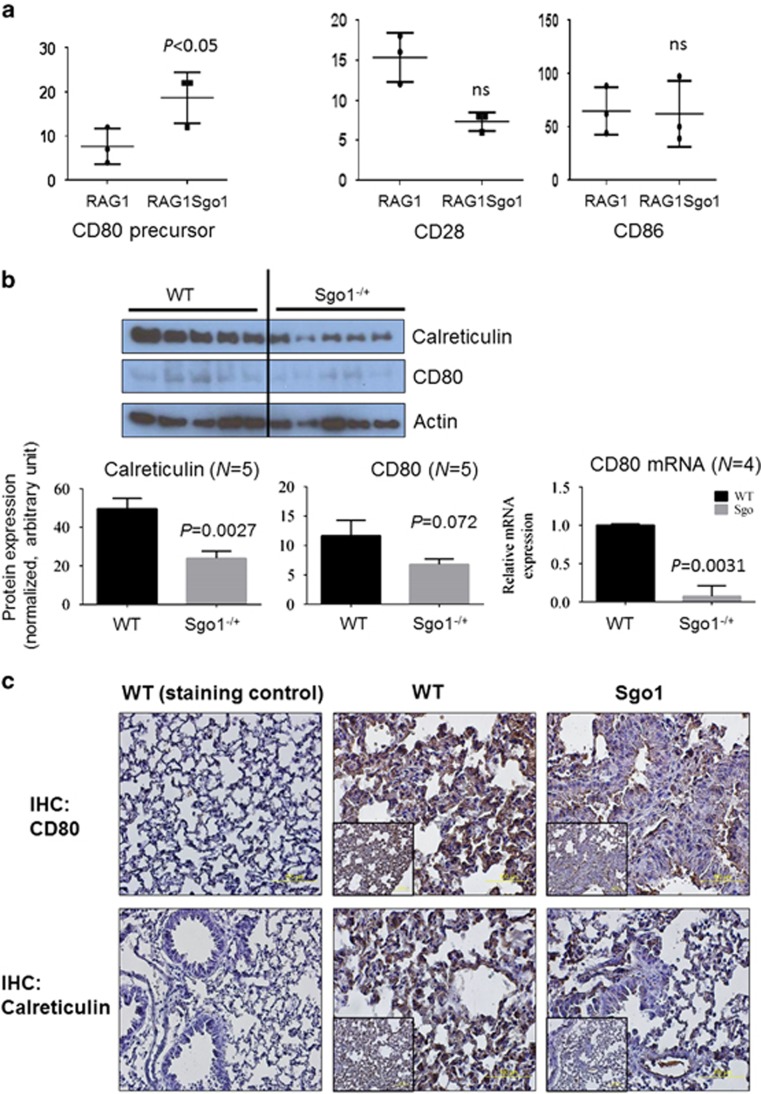

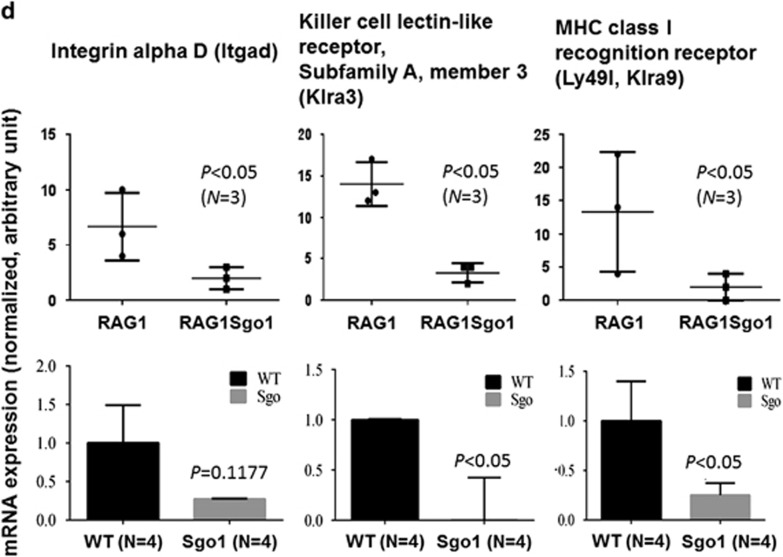
Immunomodulators for both T/B cells and NK cells were downregulated in Sgo1^−/+^. (**a**) T/B-cell activator CD80 precursor was accumulated in RAG1^−/−^Sgo1^−/+^ compared with RAG1^−/−^, suggesting a misregulation in T/B activation. There was no significant (NS) change in CD28 and CD86, which form complexes with CD80 to activate T/B cells. (**b**) In naive Sgo1^−/+^ mice, CD80 protein amount was modestly reduced and NK-cell target (the ‘eat me' signal) calreticulin was reduced, suggesting that NK-cell-mediated CIN-cell removal may be compromised. CD80 mRNA was significantly downregulated in naive Sgo1^−/+^. (**c**) Consistent with immunoblots, IHC also indicated that CD80 and calreticulin proteins were reduced in Sgo1^−/+^ with RAG1^+/+^ background. (**d**) Markers of NK-cell activation (Itgad, Klra3 and Ly49I) were also downregulated, suggesting dysfunction in NK cells in Sgo1^−/+^, both in RAG1^−/−^ and RAG1^+/+^ backgrounds.

As lung carcinomas preferentially developed on the RAG1^−/−^ background, we hypothesized that adoptive immunity is involved in suppressing CIN cells in the lung, and that genes or proteins involved in the immune system would be misregulated in Sgo1. The CD80 precursor was among the upregulated genes in RAG1^−/−^Sgo1^−/+^ (4.3-fold). CD80 and CD86 work as ligands on the surface of activated B cells and monocytes, and bind to the CD28 receptors on T cells for T-cell activation and survival. However, the RNAseq analyses showed no significant differences in CD86 and CD28 expression (Figure 5a). To test whether CD80 is misregulated in naive Sgo1, we compared CD80 protein expression in control and Sgo1^−/+^. CD80 mRNA was significantly downregulated (*P*=0.0031) and the protein was modestly reduced (Figure 5b), suggesting partial impairment of T/B-cell activation in Sgo1 without RAG1^−/−^.

As many immunomodulators are regulated post-translationally, we also tested protein expression of select immunomodulators. As RAG1^−/−^ mice depend on NK-cell function to compensate for compromised adoptive immunity, we hypothesized that NK-cell activation may also be reduced on the Sgo1^−/+^ background. Supporting this notion, protein expression of calreticulin (the ‘eat-me' signal for NK cells) was reduced (Figures 5b and c). In addition, we observed lower expression of genes indicative of NK-cell activation in the RNAseq. Integrin alpha-D (Itgad) is an adhesion molecule whose upregulation occurs in lung macrophages and is necessary for expression of TNF-alpha and recruitment of neutrophils to the lungs.^47^ Killer cell lectin-like receptor, subfamily A, member 3 (Klra3) is an NK-cell receptor that is required for activation. Major histocompatibility complex class I recognition receptor (Ly49I, Klra9) serves as an NK-cell activator^48^ and is involved in controlling cytomegalovirus infection.^49^ The reduction in receptors for NK-cell activation would explain the higher degree of lung carcinoma development in RAG1^−/−^ Sgo1^−/+^ animals, because of reduced NK-cell-mediated surveillance for mutagenic CIN cells. The reduction was confirmed in naive Sgo1^−/+^ with quantitative reverse transcriptase–PCR, and was not limited to the RAG1^−/−^ background (Figure 5d).

### Downregulations of T- and B-cell activation markers in Sgo1^−/+^ lung

**Figure 6:**
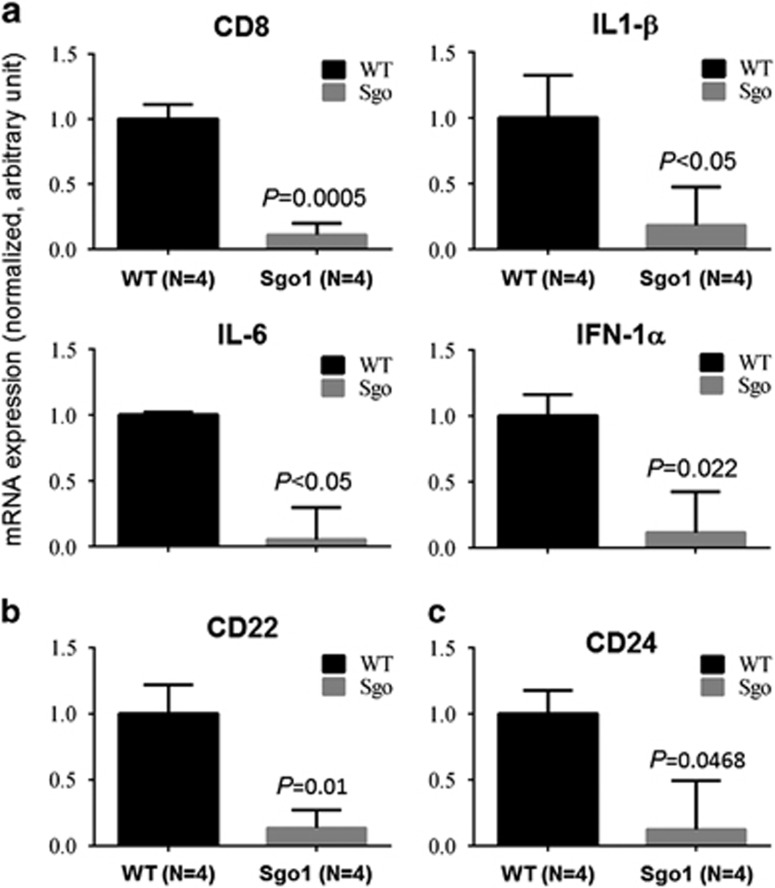
Decreases in markers for T- and B-cell activation in naive Sgo1^−/+^ lungs. Specific markers for T- and/or B-cell activation were quantified by real-time PCR in naive Sgo1^−/+^ lungs. (**a**) T-cell activation (CD8, IL-1β, IL-6, IFN-α), (**b**) B-cell activation (CD22) and (**c**) T- and B-cell activation (CD24). The activation markers all indicated significant (*P*<0.05) downregulation in naive Sgo1^−/+^ lungs.

In the above results provided, we further tested expression of markers for T-cell activation (that is, CD8, IL-1β, IL-6, IFN-α Figure 6a), B-cell activation (CD22; Figure 6b), and T- and B-cell activation (CD24; Figure 6c). They all indicated significant (*P*<0.05) downregulation in naive Sgo1^−/+^ lungs, showing an immunocompromised state.

### Cytoskeleton disturbance

Other notably downregulated genes in the RNAseq were Formin2 (4.40-fold), thymosin beta (Tb15c; 4.56-fold) and thymosin beta-like (LOC666244; 4.56-fold). All of these genes are involved in regulation of the actin cytoskeleton.

## Discussion

To suppress pre- or early-cancer cells and prevent cancers, various strategies can be utilized. These strategies include inhibition of oncogenic signaling, activation of cell death, activation of tumor suppressors that induce senescence, differentiation and/or non-proliferation, and immune surveillance.^50^ However, comprehensive pathway identification is necessary to formulate an effective strategy without an oversight to antagonizing multiple pathways. For the purpose, CIN models are uniquely valuable. Cellular level CIN, caused by environmental factors or gene mutations, can manifest locally as an initial step of carcinogenesis. The use of tissue/organ-level comparative RNAseq-transcriptomics with CIN mouse models can magnify the molecular effects that occur in cells with CIN, enabling us to identify the cellular effects of CIN in the particular organ. Thus, the use of bioinformatics with a CIN model is an effective way to identify initial molecular changes in an organ of interest.

**Figure 7:**
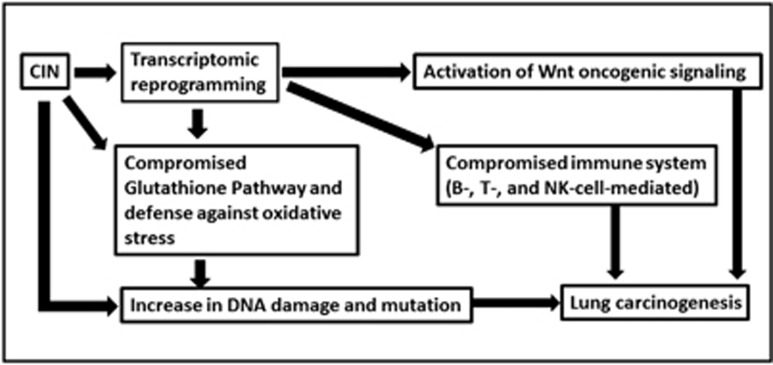
A hypothetical model for how Sgo1-CIN causes proneness to lung carcinogenesis. Sgo1^−/+^ and CIN mediate proneness to lung carcinogenesis through transcriptomic reprogramming affecting multiple pathways, including (i) Wnt activation, (ii) glutathione depletion and increased oxidative stress, (iii) DNA damage, possibly through CIN itself and oxidative stress and (iv) reduced immune surveillance. Both adoptive immunity and NK-cell-mediated cytotoxicity are decreased, and (v) cellular effects, including a disturbance in actin cytoskeleton regulators. Mechanism for the transcriptomic reprogramming warrants future investigation.

The present bioinformatic-based characterization study suggested that CIN is involved in the development of lung adenocarcinomas through the pathways newly identified in the study. We identified reprogramming in transcription in Sgo1-CIN mice lungs, leading to (1) decrease in protection by glutathione (GSTM5, glutathione pool), (2) increase in DNA damage possibly with CIN itself and with oxidative stress, (3) activation of Wnt signaling (SFRP4-β catenin-cyclinD, Lif1), (4) decrease in T/B-cell activation and immune surveillance (CD80, CD8, IL-1β, IL-6, INF-1α, CD22, CD24), (5) decrease in NK-cell activation and immune surveillance (calreticulin, Itgad, Klra3, Klra9) and (6) others such as actin cytoskeleton disturbance. All can create pro-carcinogenic lung tissue environment (Figure 7).

Aneuploidy is an outcome of CIN. We anticipated that CIN-mediated transcriptomic changes would show some similarities with those in aneuploid cells. Aneuploid cells in diverse organisms, including yeast, plants, mice and humans, showed highly related gene expression patterns that are conserved between species. In aneuploid cells, genes that were involved in the response to stress were consistently upregulated, and genes associated with the cell cycle and cell proliferation were downregulated.^17^ However, our results indicated organ-specific and nonspecific (that is, common among two or more organs) transcriptomic changes.^37^ Major nonspecific pathways that may be considered CIN signatures are involved in oxidative stress response and immune functions. We speculate that the difference between aneuploid models and CIN models is simply the characteristics of these two models. Aneuploid models carry aneuploid cells uniformly in all cells of the body, whereas CIN models can generate various outcomes after mitosis, such as apoptotic and necrotic cells, senescent cells and aneuploid cells. In CIN models, aneuploid is only one possible outcome. As a result, CIN models only occasionally carry aneuploid cells, and may do so temporarily.

In light of our results, previous results about lung tumor recurrence^41^ in inducible Mad2 overexpression mice may require re-interpretation. Thus far, cells with CIN were interpreted as a source of recurrence, and involvement of the immune system (or the lack thereof) in recurrence was not considered earlier. To capture various processes *in vivo* as a whole and to elucidate the complex competition among pro- and anti-carcinogenic events, the bioinformatic systems biology approach has demonstrated its usefulness.

Metabolic misregulation is another aspect of Sgo1-CIN our results indicated. It is consistent with reports from *Drosophila* mad2 model that CIN causes lethality with metabolic stress as well as oxidative stress,^51^ suggesting that CIN commonly affects cellular metabolism and oxidative stress pathway over various species.

Overall, our bioinformatic results indicated that CIN can influence carcinogenesis and possibly cancer recurrence through multiple pathways. The results also suggest possible countermeasures: glutathione supplementation, removal of CIN cells through targeting CIN-specific markers, Wnt signaling attenuation and immune restoration. Lung cancer is the most lethal cancer worldwide: it is predicted to claim 158 080 lives in the United States alone in 2016. ^52^ Thus, the rapid development of prevention measures is imperative. This study aided in identifying potential targets for the preventive drug development, and the results may lead to a combinatorial chemoprevention measure simultaneously targeting multiple pathways for better efficacy.

## Materials and methods

### Animals

Generation, genotyping and characterization of mouse embryonic fibroblasts (MEF) and the colonic carcinogenesis assay with Azoxymethane injections in Sgo1^−/+^ haploinsufficient mice have been described earlier.^20, 37^ RAG1^−/−^ mice were obtained from Dr Naveena Janakiram (OUHSC). All mice were maintained in the OUHSC BRC rodent barrier facility. Initially, RAG1^−/−^ mice and Sgo1^−/+^ mice were mated to generate F1; RAG1^−/+^ Sgo1^−/+^ mice, then F2; RAG1^−/−^ Sgo1^−/+^, RAG1^−/−^, Sgo1^−/+^ and RAG1^−/+^ mice that served as controls. Sample size was determined following Mead's resource equation. Group designation was used for randomization: all F2 litters were separated at the weaning (3 weeks of age) to male cage and female cage, and the cage and animals received a number. All animals were genotyped by PCR using genomic DNA from tail. After genotyping, we maintained all mice with regular diet (Purina) for 6, 9 or 12 months without any experimental treatment, then collected samples after killing at the end point based on the birth date/age. All mice were handled equally and both genders were used. Animal's genotype was not informed to the researcher(s) involved in sample collection. All mice were generated with the non-cancer-prone C57BL/6 background. All treatments complied with protocols approved by the OUHSC Institutional Animal Care and Use Committee. Samples from 12-month-old naive Sgo1^−/+^ and control wild-type mice that were used for confirmatory experiments were obtained from a previous study.^36^

### IHC and immunofluorescence

Lung tissues were fixed in 10% formalin, paraffin embedded and subjected to IHC (Histostain SP kit or SuperPicture 3rd Gen IHC kit, Life Technologies, Grand Island, NY, USA). The following primary antibodies were used at 1.0 μg/ml: anti-phospho-H2AX (γH2AX, Novus Biologicals, Littleton, CO, USA; NBP-1–19931), anti-SFRP4 (Abcam, Cambridge, MA, USA; ab154167), β-catenin (Santa Cruz Biotechnology, Dallas, TX, USA; SC-7199), anti-actin (Cell Signaling, Danvers, MA, USA; 4970), anti-calreticulin (Novus Biologicals; NB600-101) and anti-CD80 (R&D Systems, Minneapolis, MN, USA; AF740).

Four-to-six mice per strain were analyzed. At least 10 IHC images were captured from each tissue. The percentages of IHC-positive cells were calculated. Data were expressed as means±s.d., or as variances. The differences between groups were analyzed using Student's *t*-test (unpaired) with Graphpad Prism5 software (La Jolla, CA, USA). Immunofluorescence images were taken with a confocal microscope (Leica SP2 using LCS Software, Mannheim, Germany) in the OUHSC Laboratory for Molecular Biology and Cytometry Research.

### Histopathology

Histopathological characterization was performed in a blinded manner by a histopathologist, with duplicated hematoxylin/eosin-stained slides for each sample.

### Immunoblots and quantification

Our standard procedures were followed.^36^ Briefly, the lysates were aliquoted and the protein content was determined by Bradford reagent. The aliquots were stored at −80 °C. All primary and secondary antibodies were first standardized for dilution and were then used accordingly. Proteins were resolved on 8–12% sodium dodecyl sulfate–polyacrylamide gel electrophoresis, transferred to nitrocellulose membrane and probed with specific antibodies overnight at 4 °C. The membranes were washed three times with Tris-buffered saline (pH 7.4) for 15 min and were then incubated with anti-rabbit, anti-goat or anti-mouse horseradish peroxidase-conjugated secondary antibody (1:5000 dilutions in 2.5% milk) and visualized using chemiluminescence reagent (Thermo Scientific, Waltham, MA, USA). β-Actin was used as the loading control. Densitometry of various proteins and their respective loading controls from the same blot was performed using ImageJ 1.43 software (NIH, Bethesda, MD, USA). Relative optical density was calculated by dividing the densitometry of protein with that of the respective loading control.

### Next-generation sequencing/RNA sequencing

We isolated total RNA from lung tissues of RAG1^−/−^ (*N*=3) and RAG1^−/−^ Sgo1^−/+^ (*N*=3) mice. The RNA samples were submitted to the OUHSC Laboratory for Bioinformatics core facility for library construction and RNA sequencing with an Illumina MiSeq next-generation sequencer with each run generating approximately 30 million 2 × 150 bp paired end reads. The readouts were analyzed with Genesifter (Perkin Elmer, Seattle, WA, USA). Reads were normalized using mapped reads and base-lined to the data set. Analysis of differential gene expression of the normalized signal values between the control and experimental group was done using EdgeR, a moderated *t*-test, with Benjamini and Hochberg correction. *P*<0.05 and twofold expression thresholds were used to determine differentially expressed genes. Comparative RNAseq data sets were deposited to GEO-NCBI with accession number GSE84383.

### Total glutathione quantification

We used the OxiSelect Total Glutathione (GSSG/GSH) Assay Kit (Cell Biolabs Inc., San Diego, CA, usa; STA-312) and followed the instructions. OD^405^ measurements and curve plotting were performed with Clariostar (BMG LABTECH, Cary, NC, USA).

### Quantitative real-time PCR

We followed procedures described previously.^53^ Total RNA from normal and tumor samples was extracted using TRizol reagent for total cellular RNA (Invitrogen, Grand Island, NY, USA) per the manufacturer's instructions. Equal amounts of DNA-free RNA were used for reverse transcription (RT) reactions to make complementary DNA using an iScript cDNA synthesis kit (Bio-Rad, Hercules, CA, USA) per the manufacturer's protocol. Real-time PCR was carried out in a 12-μl reaction volume containing 5 μl of diluted complementary DNA (50 ng) and FastStart Universal SYBR Green master (Roche, Basel, Switzerland) and primers (Invitrogen). All PCRs were performed in a Bio-Rad iCycler iQTM5 real-time PCR detection system. The fluorescence threshold values (Ct) were calculated. Relative mRNA levels were assessed by standardization to actin or glyceraldehyde 3-phosphate dehydrogenase. Results are expressed as a relative fold difference in gene expression compared with control. Relative gene expression was calculated using the 2-ΔΔCT formula. PCR conditions were as follows: denaturation at 94 °C for 10 min, followed by 40 cycles at 95 °C for 30 s, 60 °C for 30 s and 72 °C for 45 s. All experiments were performed in triplicate.

## Figures and Tables

**Table 1:** Gross tumors in RAG1^−/−^, Sgo1^−/+^ and RAG1^−/−^ Sgo1^−/+^ mice

	*RAG1*^*−/−*^	*RAG1*^*−/−*^ *Sgo1*^*−/+*^	*Sgo1*^*−/+*^
Found dead	2 (10%)	6 (37.5%)	0 (0%)
Gross lung tumor	0 (0%)	6^a^^,^^b^ (37.5%)	3 (9.4%)
Normal-looking lung	18 (90%)	4 (25%)	29 (90.6%)
*N* (total)	20	16	32

Animal numbers for 12 months end point cohorts.

a Significantly different from RAG1^−/−^ mice by Fisher's exact test, *P*<0.004.

b Significantly different from Sgo1^−/+^ mice by Fisher's exact test, *P*<0.05.
