# Supplementary figures and images for: Systemic chromosome instability in Shugoshin-1 mice resulted in compromised glutathione pathway, activation of Wnt signaling and defects in immune system in the lung
Source: Oncogenesis. 2016 Aug 15;5(8):e256–. doi: 10.1038/oncsis.2016.56 (PMC5007830; doi:10.1038/oncsis.2016.56)

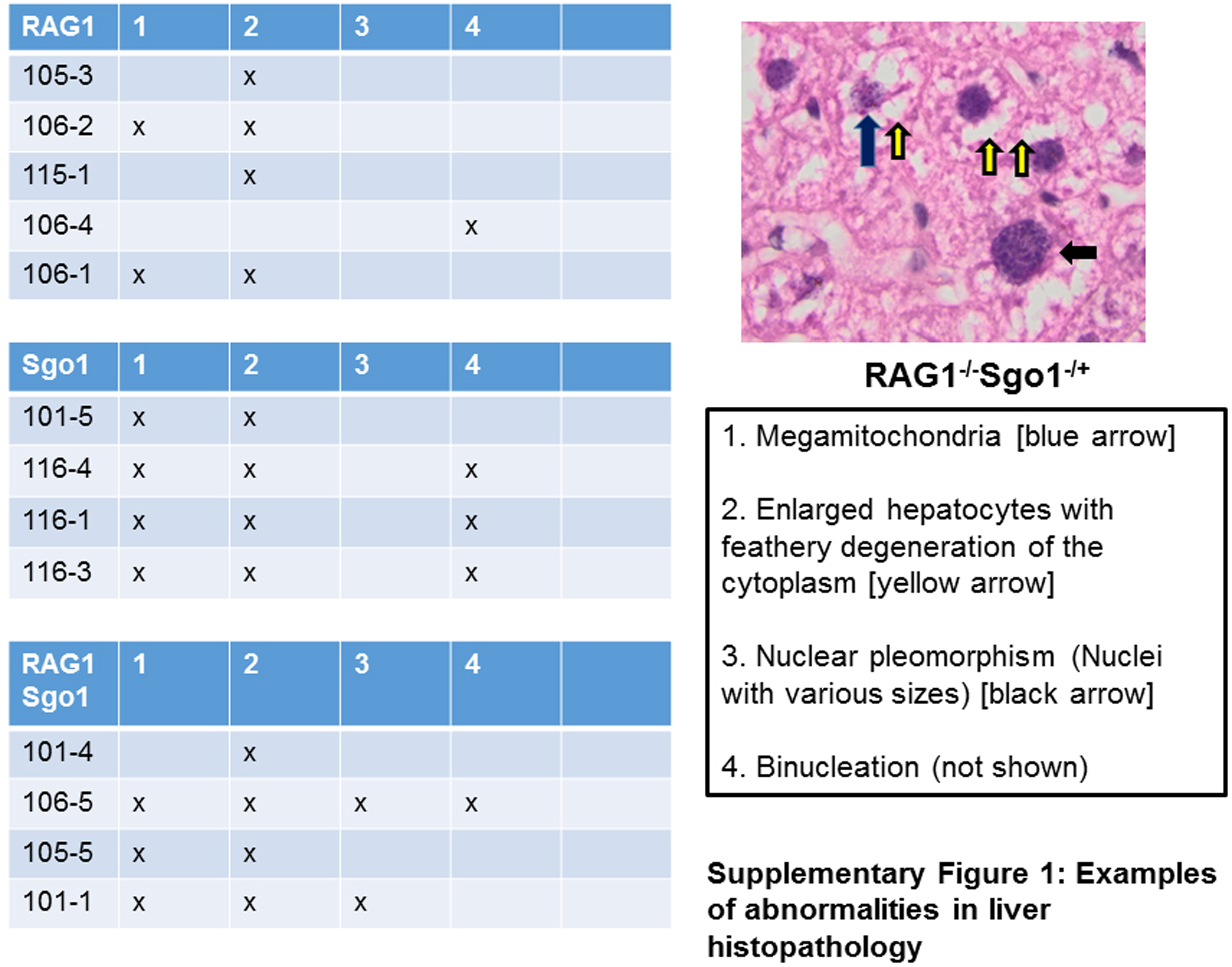

Supplement: Supplementary Figure 1 [file oncsis201656x1.tif]
